# BLV: lessons on vaccine development

**DOI:** 10.1186/s12977-019-0488-8

**Published:** 2019-10-07

**Authors:** Alejandro Abdala, Irene Alvarez, Hélène Brossel, Luis Calvinho, Hugo Carignano, Lautaro Franco, Hélène Gazon, Christelle Gillissen, Malik Hamaidia, Clotilde Hoyos, Jean-Rock Jacques, Thomas Joris, Florent Laval, Marcos Petersen, Florent Porquet, Natalia Porta, Vanesa Ruiz, Roghaiyeh Safari, Guillermo Suárez Archilla, Karina Trono, Luc Willems

**Affiliations:** 1 Estacion Experimental Agropecuaria Rafaela, INTA, 2300 Rafaela, Argentina; 2 Instituto de Virología e Innovaciones tecnológicas, Centro de Investigaciones en Ciencias Veterinarias y Agronómicas, INTA-CONICET, C.C. 1712, Castelar, Argentina; 3 Molecular and Cellular Epigenetics (GIGA) and Molecular Biology (TERRA), University of Liège (ULiège), 4000 Liege, Belgium; 4 Molecular and Cellular Epigenetics, Interdisciplinary Cluster for Applied Genoproteomics (GIGA) of University of Liège (ULiège), B34, 1 avenue de l’Hôpital, Sart-Tilman, 4000 Liege, Belgium

**Keywords:** BLV, Vaccine, HTLV, Leukemia, Retrovirus

## Abstract

Vaccination against retroviruses is a challenge because of their ability to stably integrate into the host genome, undergo long-term latency in a proportion of infected cells and thereby escape immune response. Since clearance of the virus is almost impossible once infection is established, the primary goal is to achieve sterilizing immunity. Besides efficacy, safety is the major issue since vaccination has been associated with increased infection or reversion to pathogenicity. In this review, we discuss the different issues that we faced during the development of an efficient vaccine against bovine leukemia virus (BLV). We summarize the historical failures of inactivated vaccines, the efficacy and safety of a live-attenuated vaccine and the economical constraints of further industrial development.

## Background

Bovine leukemia virus (BLV) is the etiological agent of a B-lymphocyte proliferative disease of the bovine species [1–3]. Major symptoms are lymphoma (enzootic bovine leukemia or EBL) and persistent lymphocytosis (PL) [4]. Approximately one third of BLV-infected cows will develop PL while tumors affect 5–10% of animals after long latency periods (4–10 years). At the asymptomatic stage, BLV infection is associated with reduced milk production [4], shortened longevity [5] and immune suppression [6]. Because no obvious symptoms are observed in most animals, BLV has been neglected in many regions worldwide. BLV prevalence has nevertheless a major economic impact according to recent prediction models [7]. Net benefits per cow of controlling BLV on farm is CAD 1592 for a strategy of “test and cull”. The direct impact is also associated with trade restrictions of live animals or genetic material, resulting in limitations in the access to potential markets (e.g. export from South America to EU). Death as consequence of lymphosarcoma impacts straight on the production facilities, with major losses because of no milk production, no calve replacement and costs associated with treatment and anticipated slaughter. The reduction in prevalence of 15% per year results in a positive net benefit when continued over at least 2 years [8].

With the exception of the European Union (EU), the herd prevalence of BLV worldwide ranges between 30 and 90% [8]. BLV has been eradicated from EU in the 1980′s thanks to a very expensive strategy consisting of systematic testing and culling [9]. Eradication is thus efficient but not cost-effective in highly prevalent regions. Another option is to create an independent internal facility with non-infected animals. This “test and segregate” strategy needs duplicated housing facilities and equipment in strictly separated areas [10]. This approach has been mostly unsuccessful due to increased costs and failures in long-term commitment to the program. It is also possible to take biosafety and management measures in order to minimize exposure of animals to the infectious agent. Testing and implementing best management strategies is intensively laborious, needs strict adherence to rigorous implemented measures and is susceptible to environmental factors.

Because the virion is extremely unstable, BLV transmission requires the transfer of an infected cell carrying a replication-competent provirus. Therefore, iatrogenic procedures (i.e. dehorning, ear tattooing, rectal palpation and use of infected needles) are likely the major routes of transmission. Experimental evidence and predictive models incriminate insects as potential vectors [11]. Furthermore, BLV transmission can also occur *intra utero* by a still unknown mechanism (approximately 5% calves being infected at birth). Therefore, the efficacy of a “test and manage” strategy based on strict sanitary measures has been limited.

Since BLV replication is tightly controlled by a very efficient immune response [12, 13], it should in principle be possible to select breeds that are less susceptible or even resistant to infection. Polymorphisms in major histocompatibility genes (MHC) genes have been associated with reduced proviral loads [14, 15]. However, genetic resistance to BLV infection appears to be a complex mechanism that is controlled by multiple genes. Although still unclear, the driving forces of MHC polymorphism selection may be driven by the virus itself but also by mechanisms that avoid inbreeding. Pathogen-driven selection can be based on heterozygote advantage (overdominance) or frequency-dependent selection resulting from pathogen evasion of immune recognition [16–18]. Furthermore, epigenetic mechanisms and environmental factors contribute to the outcome of infection. Therefore, it will be difficult to prioritize one allele over others as an absolute genetic selection marker for selecting BLV resistant breeds. Even more important, selection based on disease resistance may also have adverse effects on productivity traits.

Since the proviral loads are the best predictor of transmission, another strategy would consist in using antiviral therapy. Valproic acid, a lysine deacetylase inhibitor, has been successfully used to reduce proviral loads and treat BLV-induced leukemia [19]. However, long-term treatment with valproic acid is unable to eradicate the BLV reservoir and is associated with chemoresistance [20].

In this context, the availability of a safe and efficient vaccine is probably the most suitable approach to decrease prevalence of BLV worldwide.

## Why did many BLV vaccines fail?

The ideal vaccine should be safe and provide complete protection against BLV infection. It is still unclear why so many attempts were unsuccessful ([21] and reference therein). Preparations of inactivated BLV or crude lysates from persistently infected cell lines led to partial protection. Because this strategy has the intrinsic risk of transmitting infection, viral proteins, such as gp51 surface envelope glycoprotein or p24 gag antigen, were tested for prophylactic immunization. These vaccines were immunogenic but did not consistently protect from BLV challenge. Similar conclusions were obtained with short peptides, possibly due to inadequate stereochemical structure and partial epitope presentation [10]. Recombinant vaccinia viruses expressing BLV envelope glycoproteins conferred partial protection and reduced proviral loads in sheep but were unfortunately ineffective in cows. Finally, DNA vectors containing the *ENV* and *TAX* genes elicited a vigorous immune response but did not prevent later infection. As other previously developed immunogens, DNA vaccines were thus also disappointing.

In fact, available vaccines against retroviruses are extremely limited with a few marked exceptions (e.g. feline leukemia virus, FeLV). A major challenge in anti-retroviral vaccination is that, once established, the virus cannot be cleared from the host. Therefore, only a prophylactic vaccine providing sterilizing immunity represents a conceivable solution for BLV-infected animals. The criteria required to achieve this optimal vaccine are unknown but should in principle involve humoral, cytotoxic and perhaps innate immunity. The colostrum that the calf suckles soon after birth contains neutralizing anti-BLV antibodies that protect against a series of agents including BLV [10]. A strong humoral immunity is nevertheless not sufficient to provide protection since vaccines eliciting high anti-BLV antibody titers are inefficient (reviewed in [22]). Unmet criteria such as the quality of the antiviral antibodies (i.e. neutralizing activity, conformation, isotype, avidity) likely explain failure of vaccines based on inactivated viral particles, crude lysates, purified antigens and peptides. The main limitations of these vaccines include the fast decline of protective antibody titers and poor stimulation of cytotoxic response. For still unclear reasons, eliciting both humoral and cell-mediated immunity may also be insufficient as illustrated by the inability of plasmid and recombinant vaccinia virus vectors expressing BLV antigens to protect against infection [10, 11, 23, 24].

Together, these failures to obtain an efficient vaccine indicate that protection against BLV infection requires stimulation of humoral and cytotoxic immunity at different levels: quantitative (e.g. antibody titers, number of CTLs) and qualitative (e.g. type of epitope, neutralizing activity, persistence). We think that failures to obtain a vaccine result from the inadequate equilibrium between these parameters.

## An efficient vaccine against BLV is available

As would have said Thomas Edison, these numerous attempts were not failures but rather successful discoveries of “not making a good vaccine”. Therefore, we reasoned that the vaccine eliciting the best antiviral response would be the virus itself. Our data indeed indicated that it is extremely difficult, if not impossible, to infect a BLV-infected animal with another BLV strain [25, 26]. Since the BLV sequence variability within an infected animal and among strains worldwide is very limited [27], antigenic drift should not be a major issue, as observed in HIV [28, 29]. The key issue then lies in finding the right combination of deletions and mutations that would inactivate the pathogenic activity of the attenuated vaccine without loosing immunogenicity. This quest took us some time (i.e. since 1993) and involved the synergy between two complementary teams interested in basic science and having veterinary expertise. We designed an approach based on a live-attenuated BLV strain harboring multiple deletions and mutations. The rationale was to delete pathogenic genes (i.e. the oncogenic drivers, such as TAX and G4 [30]) while maintaining a low level of infectivity. After a series of failures, we have identified a deleted BLV provirus that is infectious in cattle but replicates at very low levels. Inoculation of this vaccine elicits a vigorous anti-BLV immune response comparable to that of a wild-type infection (manuscript in preparation and patent # WO2014/131844). The vaccine is currently used to vaccinate against BLV infection in commercial herd settings. Besides efficacy, the major challenge is safety of the vaccine: transmission from cow-to-calf, recombination with endogenous viruses, milk and meat composition.

## What are the issues of using an attenuated BLV vaccine?

The attenuated vaccine has been obtained by targeted mutations and deletions of an infectious BLV provirus. Therefore, it is possible that the vaccine strain undergoes genetic drift with reversion of inactivating mutations back to the wild type sequence. Another possibility is antigenic shift resulting from recombination with a wild type virus. These sequence diversifications are in fact not really problematic because the resulting strain would acquire a wild-type genotype. This situation is thus identical to a failure of vaccination. A more severe hazard is the acquisition of mutations that increase pathogenicity such as N230E substitution of an envelope N-linked glycosylation site [31, 32]. Since this mutation has never been identified in any available sequence worldwide, we think that this scenario is unlikely. Perhaps the most significant hazard is recombination with another virus or host sequence. Since genes have been deleted in the BLV vaccine, additional sequences can indeed be packaged into the virion, as observed in Rous Sarcoma virus [33]. Rare cases of recombination between the poliovirus vaccine and coinfecting enteroviruses led to reversion to a pathogenic state [34]. There are also concerns about possible activation of endogenous retroelements by the vaccine strain. Whether this potential risk is a hazard evolving into a threat will require large-scale vaccination trials. Ongoing experiments indicate that the vaccine does not undergo any genetic drift and/or shift (manuscript in preparation). It should be mentioned that these recombination events occurred in cell cultures containing high virus titers. Therefore, the risk can be reduced by using GMP-purified DNA from safe plasmid vectors based for example on the *ccd* toxin/antitoxin system [35]. Because of production costs, we are presently favoring an approach based on a stable cell line carrying an integrated vaccine. This cell system has the additional advantage to be devoid of any vector sequence but should be carefully screened for contamination by any potential pathogens.

Another risk is spread of the attenuated vaccine to non-infected animals in the herd, from the cow to their calf (e.g. secreted in milk) or from bull to heifer (i.e. semen). Ten years follow-up of uninfected sentinels did not reveal a single event of transmission during vaccination trials. Passive antibodies are nevertheless transmitted from the vaccinated cows to the newborn calves via the maternal colostrum. Although the mechanism is still unknown, it is likely that the attenuated virus is unable to spread because of limited replication capacity. Indeed, only animals with high proviral loads seem able to transmit BLV [36]. Current data indicate that the proviral loads of the attenuated vaccine even decrease progressively over time. Importantly, all vaccinated animals were protected against infection and therefore did not develop tumors.

Since BLV also infects other bovines (*zebu*, *water buffalo*) and can also be experimentally transmitted to sheep, goats or alpaca (*Vicugna pacos*), the impact of vaccination on other species should be considered. How might evolution of the virulent BLV strains be driven after widespread use of a BLV vaccine? Would the spread of the vaccine strain to wild animals cause any concern? What is the risk for recombination of the vaccine with wild type virus?

An additional issue that is possibly associated with an attenuated vaccine pertains to exhaustion. Could continuous viral expression at extremely low levels combined with a strong immune response be problematic? This mechanism would indeed lead to cell exhaustion in vaccinated animals. Notwithstanding, this issue is expected to be less tricky in vaccinated cows since the amount of viral antigen is lower compared to animals infected with wild-type virus.

Is BLV zoonosis an issue? Although controversial, recent reports suggest that BLV may be associated with human cancer [16]. To be demonstrated, the link between BLV and human cancer would require further functional and epidemiological evidence. Only a few studies have investigated a possible link between dietary exposure to BLV and human cancer [37–40]. Contradictory conclusions were drawn because these studies were not designed prospectively to specifically address the association between dairy consumption and cancer. The most striking functional evidence was provided by onset or erythroleukemia in chimpanzees fed with BLV-infected milk [41–43]. If the association is demonstrated beyond correlation studies, the dilemma would be to choose between hazards linked to large-scale vaccination and a threat of breast cancer in regions where BLV is highly prevalent. Since vaccination is expected to reduce prevalence, elimination of BLV in cattle would be promoted. Our data also indicates that the vaccine is not present in milk and meat, suggesting that bovine-derived food would be less hazardous.

## What are the lessons for HTLV vaccination?

Some aspects of vaccination against BLV may be instructive for the design of a vaccine against HTLV. Important questions relate to the type of vaccine, the target population, the modes and the goals of vaccination.

At first glance, the option of an attenuated vaccine would not be considered because of potentials hazards that are not justified by expected gains. Indeed, a probability of 5% to develop disease, either tropical spastic paraparesis/HTLV-associated myelopathy (HAM/TSP) or Adult T-cell leukemia/lymphoma (ATLL), does not justify the risk. The failures of BLV vaccines based on purified proteins, peptides, inactivated antigens or recombinant vector vaccines indicate that the situation is more complex than expected. However, design of a vaccine against HTLV will nowadays benefit from the most recent developments in terms of vectorisation, antigen selection, purification and combination with optimal adjuvants. It should nevertheless be mentioned that inactivated or subunit vaccines are not devoid of risk as illustrated for FeLV [44] and other viruses [45]. Although still obscure, the mechanism involves antibody-dependent enhancement of viral infection. A number of unsuccessful trials in the BLV model further indicate that an efficient vaccine requires a subtle qualitative and quantitative equilibrium of humoral and cytotoxic immunity. These characteristics are clearly more difficult to fulfill for inactivated or subunit vaccines.

Should the HTLV vaccine be prophylactic, therapeutic or both? These options have their specific requirements and limitations. A large-scale preventive vaccination as we propose for BLV is probably not justified due to the low prevalence of HTLV in many regions worldwide. A cost/benefit evaluation should be undertaken to identify the target population. It is nevertheless predicted that prophylactic vaccination would be beneficial in endemic regions such as Australia or Japan [46]. Since colostrum antibodies protect against infection, should pregnant and/or breastfeeding mothers also be vaccinated? Besides, vaccination of children from HTLV-infected mothers may be impaired by colostrum intake, as we encountered in the BLV model. In this context, the age of vaccination may also be crucial because of a potential risk of autoimmunity. It would be interesting to address this mechanism upon BLV vaccination since immunity is still immature at birth in the bovine species.

As indicated earlier, only prophylaxis preventing infection is useful for BLV. For HTLV, therapeutic vaccines may boost antiviral response and improve disease outcome by tempering morbidity of HAM/TSP and increasing survival in ATLL. It remains nevertheless possible that vaccination with viral antigens such as TAX and HBZ would rather activate viral replication [47]. Other risks include antibody-dependent enhancement of viral replication. In this context, the BLV system could provide a model to address specific questions to advance in HTLV vaccine development, in particular safety risks (recombination with endogenous sequences, side effects, viral transmission and pathogenesis).

## Conclusion

We have developed a vaccine against BLV using a strain that lost pathogenic potential while remaining sufficiently antigenic to induce lasting protective immunity. Obtaining a vaccine providing sterilizing immunity has been a long story requiring bypass of a number of hurdles. The BLV paradigm has illustrated that vaccine development is possible and may be a model for viruses in other species (e.g. HTLV). Current efforts aim at making this vaccine available worldwide. After having bypassed most technical hurdles, the challenge is now to achieve industrial scale-up, local registration of the vaccine and approval by the end users. Perhaps the most important risk is the lack of interest of the industry that only focuses on high profit developments. This is unfortunately also true for a future HTLV vaccine.

## Data Availability

Not applicable

## Abbreviations

ATLL: adult T cell leukemia/lymphoma
CTL: cytotoxic T cell
BLV: bovine leukemia virus
EBL: enzootic bovine leukemia
PL: persistent lymphocytosis
EU: European Union
FeLV: feline leukemia virus
HAM/TSP: tropical spastic paraparesis/HTLV-associated myelopathy
